# Analysis of Complete Puumala Virus Genome, Finland

**DOI:** 10.3201/eid1812.120747

**Published:** 2012-12

**Authors:** Angelina Plyusnina, Maria Razzauti, Tarja Sironen, Jukka Niemimaa, Olli Vapalahti, Antti Vaheri, Heikki Henttonen, Alexander Plyusnin

**Affiliations:** Author affiliations: University of Helsinki and HUSLAB, Helsinki, Finland (A. Plyusnina, M. Razzauti, T. Sironen, O. Vapalahti, A. Vaheri, A. Plyusnin);; Finnish Forest Research Institute, Vantaa, Finland (J. Niemimaa, H. Henttonen)

**Keywords:** HFRS, nephropathia epidemica, hantavirus, Puumala virus, viruses, Finland, zoonoses, rodents

## Abstract

Puumala virus causes nephropathia epidemica, a rodent-borne zoonosis that is endemic to Europe. We sequenced the complete Puumala virus genome that was directly recovered from a person who died and compared it with those of viruses from local bank voles. The virus strain involved was neither a unique nor rare genetic variant.

The outcome of a viral infection is determined by the agent’s pathogenicity and by host factors, such as genetic predisposition. For RNA viruses, which are notorious for their swift evolution and adaptation, a pathogen’s specific genotype usually is to blame for devastating effects (*1*). In many cases, however, the virus genome is not easy to search for particular mutations because recovery of complete viral sequences from clinical specimens remains extremely difficult. This is especially true for hantaviruses (family *Bunyaviridae*) that cause hemorrhagic fever with renal syndrome (HFRS) and hantavirus cardiopulmonary syndrome (*2*,*3*). Thus far, only a few complete hantavirus genomes originating from persons with clinical cases have been reported (*4*,*5*), and only 1 was recovered without passaging first in cell culture (*4*), which by itself can induce adaptive changes in the viral genome (*6*). We present the complete genome of PUUV directly recovered from a person with fatal infection.

Usually PUUV causes mild HFRS (also called nephropathia epidemica [NE]). In Finland, 1,000–3,000 NE cases are diagnosed annually, i.e., ≈60 cases/100,000 persons during years when the vole population peaks (*7*). Almost 100% of infected persons recover, and long-lasting complications are rare. The few fatal cases reported (*8**,**9*) showed no apparent geographic clustering. Thus, whether more severe illness could be connected to certain genetic variants of PUUV remains unknown.

## The Study

The patient was a previously healthy 37-year-old man with a history of smoking. He died in November 2008 of severe NE on day 4 after the onset of symptoms that started with high fever, vomiting and diarrhea, headache, and visual disturbances. His condition deteriorated quickly, and multiorgan failure developed, including respiratory distress, acute kidney failure, liver failure, and severe thrombocytopenia.

A standard autopsy was performed, and tissue samples were stored fresh at −70°C and fixed in formalin. PUUV infection was confirmed initially by IgM test and later by reverse transcription PCR (RT-PCR), followed by sequencing. Genetic analysis was performed from autopsy samples stored fresh at −70°C (the high quality of clinical samples was crucial for the downstream applications). Complete PUUV genomes were recovered in a set of nested and seminested PCR (sequencers of primers are available on request). Amplicons were gel-purified and sequenced directly by using ABI PRISM Dye Terminator sequencing kit (PerkinElmer/ABI, Foster City, CA, USA). Quantitative RT-PCR was used to measure PUUV load with DyNAmo Capillary SYBR Green kit (Finnzymes, Espoo, Finland). Copy numbers were calculated from a standard curve created by using in vitro transcribed PUUV small (S) segment RNA (T7 transcription kit, Fermentas, Vilnius, Lithuania).

**Figure 1:**
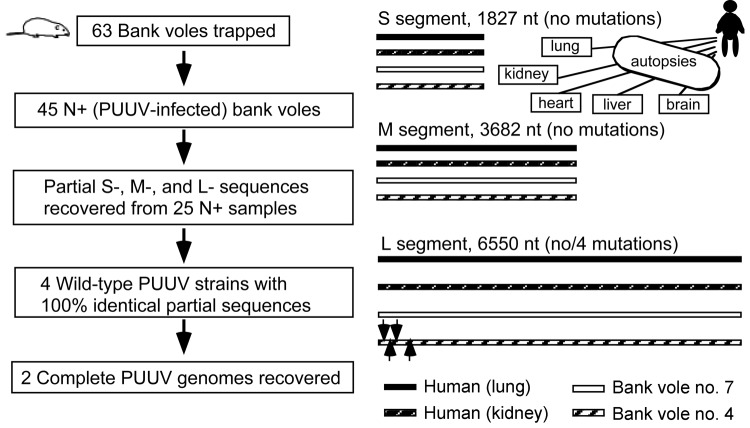
Comparison of PUUV-genome sequences recovered from human autopsies and rodent tissues. Locations of 4 silent mutations found in the L-segment sequences: G114A, U261C, A349G, and U378A are indicated by arrows (right column). PUUV, Puumala virus; S, small; M, medium; L, large; +, positive.

Quantitative RT-PCR revealed the highest numbers of virus genome copies in lungs and kidneys: 1,881 and 1,136 per μg of total RNA, respectively. Copy numbers per μg of total RNA in other tissues were lower: 240 in the heart, 160 in the spleen, 50 in the liver, and 42 in the brain. In agreement with these findings, complete PUUV genome sequences (12,059 nt) were recovered from the lung and kidney and partial sequences of different lengths from heart, liver, and brain (Figure 1). Corresponding sequences recovered from different tissues were identical, i.e., no tissue-specific mutations were observed.

To determine whether this fatal NE case was caused by an unusual or rare genetic variant of PUUV, we searched for identical or closely related genetic variants in bank voles trapped near the patient’s house (storage buildings and surroundings within 500 m) in Pieksämäki, central Finland (62°18′N, 27°08′E). Travel history of the case-patient suggested that the infection had been acquired at his residence. In 2008, the vole population peaked in the southern half of Finland, including Pieksämäki, and 3,259 NE cases were diagnosed nationwide (*7*), the highest number ever registered in Finland. Sixty-three bank voles were snap-trapped during 3 consequent nights in December 2008.

Lung tissue samples from the bank voles were screened for PUUV N protein antigen by using immunoblotting, and 45 (71%) voles tested positive. Tissues from 25 virus-infected voles were taken for genetic analysis, and partial sequences of PUUV genome S, medium (M), and large (L) segments (≈12% of the total virus genome) were recovered from them.

**Figure 2:**
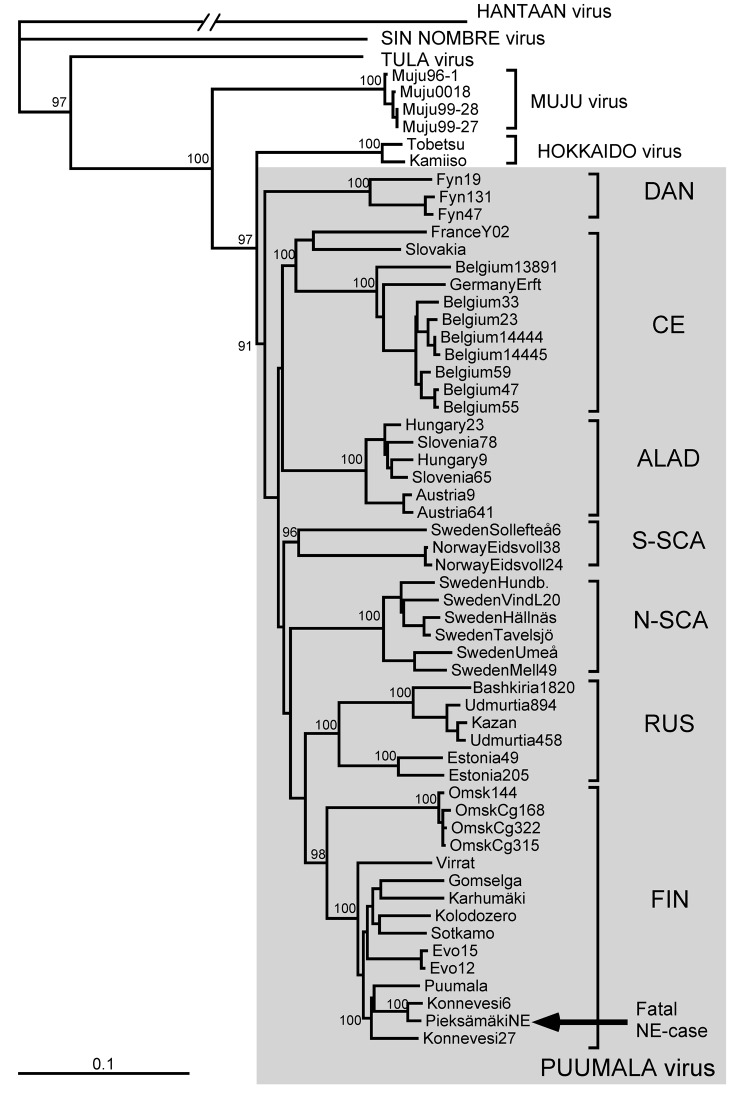
Phylogenetic tree of PUUV S segment sequences (coding region). Topologies of the M and L trees were similar (not shown). Calculations were performed by using the PHYLIP program package (distributed by J. Felsenstein, University of Washington, Seattle, WA, USA). Five hundred bootstrap replicates were generated by using the SeqBoot program and submitted to the distance matrix algorithm (DNAdist program), with the maximum-likelihood model for nucleotide substitutions). The resulting distance matrices were analyzed by using the neighbor-joining tree-fitting algorithm (Neighbor program). The bootstrap support values were calculated by using the Consense program. Hantavirus sequences used for comparison were recovered from GenBank. Gray shading indicates PUUV strains. PUUV, Puumala virus; S, small; M, medium; L, large; DAN, Danish; CE, Central European; ALAD, Alpe-Adrian; S-SCA, South Scandinavian; N-SCA, North Scandinavian; RUS, Russian; FIN, Finnish; NE, nephropathia epidemica. Scale bar indicates genetic distance of 0.1.

In agreement with previously published data (*10*), the number of PUUV genome copies in bank vole tissues was within the range of 10^5^–10^6^/μg of total RNA, i.e., ≈100-fold higher than in tissues of the case-patient. Partial virus sequences from 4 voles were 100% identical to those from human tissues. Next, complete PUUV genome sequences were recovered from 2 of these voles; the sequences differed at only 4 positions in the L segment (all silent mutations; Figure 1, right column). One of the complete rodent-originated PUUV sequences was 100% identical to the sequence from the case-patient. PUUV sequences have been deposited in GenBank under accession nos. JN831943–JN831952. Phylogenetic analysis confirmed that the hantavirus involved belonged to *Puumala virus* species and was most closely related to the earlier described genetic variants from Finland, particularly to those circulating at Konnevesi (62°34′N, 26°24′E) and Puumala (61°52′N, 28°17′E) localities (Figure 2).

## Conclusions

Our findings established an unequivocal genetic link between the fatal human NE case and local wild-type PUUV strains. These findings also revealed that no mutations had accumulated in the genome of PUUV during transmission of the virus to the patient and the fatal generalized infection that followed. Finally, we demonstrated that the wild-type PUUV strain that caused the fatal infection was neither a unique nor rare genetic variant; the exact sequence match to the complete human-originated PUUV sequence was found among the first 25 bank voles analyzed. Genetic links of the type have been reported for PUUV infections in Finland (*11*) and for Sin Nombre virus infection during the outbreak in the Four Corners area of the United States (*4*,*12*), but perfect sequence match was not observed.

In PUUV infections, renal insufficiency is a hallmark of the disease, but pulmonary, cardiac, central nervous system, ocular, and hepatic manifestations and, in severe cases, hypophyseal injury also can occur (*13*). In the fatal case described here, death resulted from multiorgan failure when kidneys, lungs, heart, and liver were affected. The viral load was higher in the lungs and kidneys and lower in the heart, spleen, liver, and brain. Whether this load distribution is unique for fatal PUUV infections remains to be seen because corresponding data for other hantavirus infections are missing. Moreover, severe histopathologic changes were detected not only in lungs and heart but also in liver and hypophysis, whereas kidneys, in this respect, were almost normal. Thus, viral load does not seem to correlate with tissue pathology. A more detailed pathologic description of this and other lethal cases is under way.

Two more observations might be relevant to the case. First, human leukocyte antigen typing showed that the patient had the risk haplotype for severe NE including a C4A null allele, i.e., a major antivirus defense system complement was impaired (T. Sironen et al., unpub. data). Second, the patient was a smoker and thus more likely to become infected with PUUV (*14*). These factors might have substantially affected the fatal outcome. We anticipate that our investigation will prompt further full-length genome analyses of the wild-type strains of bunyaviruses that cause infections in humans.
